# Long-Term Follow-up of a Randomized Study of Oral Etoposide versus *Viscum album* Fermentatum Pini as Maintenance Therapy in Osteosarcoma Patients in Complete Surgical Remission after Second Relapse

**DOI:** 10.1155/2020/8260730

**Published:** 2020-04-26

**Authors:** Alessandra Longhi, Marilena Cesari, Massimo Serra, Erminia Mariani

**Affiliations:** ^1^Chemotherapy Division, IRCCS Istituto Ortopedico Rizzoli, Bologna, Italy; ^2^Laboratory of Experimental Oncology, IRCCS Istituto Ortopedico Rizzoli, Bologna, Italy; ^3^Immunoreumathology and Tissue Regeneration Laboratory, IRCCS Istituto Ortopedico Rizzoli, Bologna, Italy

## Abstract

**Background:**

In relapsed osteosarcoma, the 5-yr postrelapse disease-free survival (PRDFS) rate after the second relapse is <20%. In June 2007, a randomized study was started comparing oral etoposide vs *Viscum album* fermentatum Pini (an extract derived from the parasitic plant *Viscum album* L., European mistletoe) as maintenance therapy in patients with metastatic osteosarcoma in complete surgical remission after the second relapse. The primary endpoint was the PRDFS rate at 12 months (compared to the historical control rate). This is a long-term updated result. *Patients and Methods*. 10 patients received oral etoposide 50 mg/m^2^ daily for 21 days every 28 days for 6 months, and 9 patients received *Viscum album* fermentatum Pini 3 times/wk subcutaneously for 1 year. The study closed early in July 2011 due to insufficient recruitment. Lymphocyte subpopulations were analyzed at T0, T3, T6, T9, and T12 months.

**Results:**

On 30 June 2019, at a median follow-up ITT of 83 months (range 3–144 ms), a median PRDFS of 106 ms (2–144) was observed in the *Viscum* arm with 5/9 patients who never relapse vs a PRDFS of 7 months (3–134) in the etoposide arm (all patients in the Etoposide arm relapsed) (hazard ratio HR = 0.287, 95% CI: 0.076–0.884, *p*=0.03). Model forecast 10-yr overall survival rates as 64% in the *Viscum* arm and 33% in the etoposide arm. Lymphocyte subpopulation counts (CD3, CD4, and CD56) showed an increase in the Viscum arm while a decrease was observed in the etoposide arm during treatment.

**Conclusions:**

After 12 years from the start of the trial, the patients in the *Viscum* arm continue to show a considerably longer PRDFS compared to oral etoposide, and a trend for an advantage in OS is evident even if the number of treated patients is too small to draw conclusions. *Viscum* as maintenance treatment after complete surgical remission in relapsed osteosarcoma should be further investigated and compared with other drugs.

## 1. Introduction

Overall survival of patients with relapsed osteosarcoma remained unsatisfactory and unchanged over the last three decades, as well as the efforts to develop novel active agents have generally yielded disappointing results [1,2]. Main prognostic factors for the survival of relapsed patients include the location of relapse (better prognosis for the lung vs bone), the time to progression (>24 months vs shorter periods), and the number of lung metastases (1–3 vs more than 3 nodules) [3]. Relapse mainly affects the lungs, and patients achieving complete surgical remission after relapse have a prolonged 5-year postrelapse survival [1]. Subsequent repeated relapses decrease further the chance of cure. In our previous study, 235 relapsed osteosarcoma patients had a 5-yr PRDFS of 29%, and only 14 out of 120 (11.6%) patients who had a second relapse never relapsed for the third time [1]. The COSS study published in 2009 on 249 osteosarcoma patients after a second relapse showed that the five-year overall and event-free survival rates were 16% and 9%, respectively [2]. A more recent paper (2017) [4] on 60 Italian patients with relapsed osteosarcoma followed from 2003 to 2013 showed that the median postrelapse disease-free survival (PRDFS) after the second relapse was 6 months (range 42 days to 44 months). The majority of patients (84%) relapsed less than 12 months after the second complete surgical remission. The 5-year postsecond relapse survival rate was 22%. Lung recurrence as a unique site correlated with a better 5-year survival (33.6%) compared to other sites of recurrence (5%; *p*=0.008) [4]. Patients who relapse with operable metastases are usually operated. Postoperative adjuvant chemotherapy is usually not a standard after relapse because there is no evidence of an advantage that would outweigh the chemotherapeutic toxicity, and most effective drugs are employed in the adjuvant phase. Chemotherapy in a relapsed patient is usually administered in advanced or inoperable diseases. Tumors have immunosuppressive capacities through secretion of various cytokines favoring an escape from the immune response [5]. Therapeutic strategies to counteract the tumor-induced immunosuppressive effect are employed by blocking immune checkpoints (CTLA-4, PD-L1, or PD-1) on T cells with specific antibodies and thereby restoring the cytotoxic capacity of these cells [6]. Immunotherapy in bone sarcoma was first applied by Coley who injected a mixture of streptococcal bacteria into unresectable bone sarcomas in 1891, achieving an immunological reaction and tumor regression [7]. Few studies are available on immunotherapies in osteosarcoma with limited results [8]. Interferon proved to be unsatisfactory in improving the overall survival in adjuvant setting in localized osteosarcoma in the EURAMOS 1 trial [9]. Muramyl tripeptide (MTP), a BCG-derived drug, which activates macrophages, administrated together with ifosfamide, improved 6 years of OS from 70% to 78% (*p*=0.03) in patients with localized osteosarcoma in adjuvant setting [10]. MTP has been licensed in European countries for the adjuvant treatment of localized osteosarcoma.

Immunotherapy with anti-PD-L1 showed promising cure advantages in some cancers other than osteosarcoma. Less than 20% of osteosarcoma patients are PD-L1 positive. A recent report of pembrolizumab (an anti-PD-L1) employed in a 12 advanced osteosarcoma patients series produced unsatisfactory results [11].

*Viscum album* extracts (European mistletoe) is part of integrative medicine, and its usage is popular among cancer patients in German-speaking countries [12,13]. *Viscum album* extracts contain a variety of immunoactive compounds that include lectins, viscotoxins, oligosaccharides and polysaccharides, flavonoids, and triterpene acids [14]. The antitumor activity of *Viscum* lectins has been demonstrated both in *in vitro* and *in vivo*, and it has been supposed to be related to the activation of monocytes/macrophages, granulocytes, natural killer cells, T cells, and dendritic cells and to the induction of a variety of cytokines [5]. Furthermore, *Viscum album* extracts appear to interfere with tumor angiogenesis [15].

Recently, xenograft models showed the activity of a *Viscum* extract in osteosarcoma [16] and in Ewing sarcoma both in *in vitro* and *in vivo* conditions [17]. In analogy to previous studies on other tumors, it was shown that *Viscum* extracts have proapoptotic activity on osteosarcoma cells, via caspase activation, and also it displayed a synergistic activity with chemotherapeutic drugs usually employed in osteosarcoma therapy (doxorubicin, ifosfamide, and etoposide) [16]. A further preclinical study demonstrated the cytotoxic activity of *Viscum* Species in different pediatric cancer cells [18].

Other *in vitro* studies demonstrated different activation of dendritic cells and promotion of Th1 immune response according to different species of *Viscum* [19].

Some therapeutic protocols for osteosarcoma employed etoposide (topoisomerase II inhibitor) mainly IV in combination with ifosfamide at relapse. Yet, oral administration is used as well, and in clinical practice, usually in recurrent disease. A recent report [20] on 28 metastatic osteosarcoma patients showed that the use of etoposide 25 mg t.i.d induced 15% partial remission. The median PFS was 3.7 months, and the median overall survival was 7.4 months.

Hematologic toxicity is one of the main limiting characteristics of etoposide with an increased risk of secondary malignancy either after IV [21] or after oral administration [22–24].

In a previous paper [23], we presented the results of a randomized study of a *Viscum album* extract or oral etoposide administration in osteosarcoma patients in complete surgical remission after second relapse. In which, PRDFS rates, quality of life, and tolerability of each therapy after 12 months of treatment were reported [25].

In the present paper, we report updated results on PRDFS and on overall survival (OS) of those study population after a follow-up period of 12 years from the opening of the study.

## 2. Materials and Methods

This monoinstitutional, prospective, randomized, open-label study approved by the Ethic Committee of the Istituto Ortopedico Rizzoli (IOR), Bologna, was registered in the in the EU clinical trials register (EudraCT number 2006-002676-18) and conducted according to the Declaration of Helsinki. Patients in complete surgical remission after the second relapse for osteosarcoma were enrolled starting in June 2007, and 18 out of 20 patients had lung metastasectomy for their second relapse; lung metastases were 1–3 nodule each and were all resected in our institute from the same team of surgeon, and margins were considered wide if occurred in normal lung tissue. Two patients had bone metastases, and their margins were wide.

Study characteristics are presented in summarized form only. For a detailed description of the whole study and of patients' inclusion criteria, sample size, and patients' randomization see Longhi et al. [25].

### 2.1. Treatment

Etoposide was administered orally in doses of 50 mg/m2 per day for 21 days every 28 days for 6 cycles. *Viscum album* fermentatum Pini (manufactured as “Iscador P” by Iscador AG, Switzerland) was administered subcutaneously 3 times a week for 12 months. The *Viscum album* extract was injected subcutaneously (abdominal) 3 times/week. The starting dose was 2 boxes of Series 0 for 14 vials (each box contains 2 vials of 0.01 mg, 2 vials of 0.1 mg, and 3 vials of 1 mg), followed by 2 boxes of series I for 14 vials (2 vials of 0.1 mg, 2 vials of 1 mg, and 3 vials of 10 mg) vials, and subsequent treatment with series II (1, 10, and 20 mg) was carried out continuously until the 12^th^ month [25].

### 2.2. Clinical Assessment

Clinical and radiological assessment was done at the screening visit and after 3, 6, 9, and 12 months [25] of treatment, and then if no progression was registered, it was done every 4 months until 3 years, then every six months until the 5^th^ year, and then every year for at least until the tenth year. All patients were also planned to undergo blood test for the evaluation of lymphocyte subpopulations at T0, T3, T6, T9, and T12 months. Phenotype characterization of the different lymphocyte subpopulations was performed by specific monoclonal antibodies (CD3 = panT, CD4 = helper, CD8 = suppressor, CD56 and CD16 = natural killer, and CD19 = panB).

### 2.3. Statistical Methods

For the long-term evaluation presented here, PRDFS and OS have been statistically compared between treatment arms using the log-rank test. Hazard rates and 95% confidence intervals (CI) have been derived from a corresponding Cox proportional hazard regression with the treatment arm as the only independent factor. Forecasts of 10-year OS rates have been estimated using parametric proportional hazard regression models with lognormal, exponential, log-logistic, or Weibull distribution of the survival times (Klein and Moeschberger, 1997). The lognormal model fitted the data best (smallest value for −2∗log-likelihood); therefore, these estimates are shown. Due to the decrease in sample size from 18 to 10 per study arm, no other adjustments were done, especially the global alpha error level of 5% remained unchanged in order to not violate the validity of the statistical testing procedure and its interpretation. Due to the decrease in sample size from 18 to 10 per study arm, the power of the single-arm binomial test for superiority of a given treatment over the historical PRDFS rate of 12% dropped from 81% to 61.8%. However, once a statistically significant difference (here, the superiority of 55.6% PRDFS rate of the *Viscum* arm at 1 yr over the historical 12% rate) has been found, the power of the (planned) test becomes irrelevant (i.e., statistically significant difference is valid independent on sample size). With regard to etoposide, the 1-yr PRDFS was 27.3% compared with the expected rate of 35% which is not statistically significant. Anyway, the long-term follow-up revealed a PRDF totally significant for *Viscum* over the etoposide arm.

## 3. Results

From June 2007 to July 2011, twenty patients were randomized. Eleven patients were randomly assigned to the etoposide arm and nine to the *Viscum* arm. One patient enrolled in the etoposide arm refused to accept etoposide after randomization, and he has withdrawn from the trial; he was analyzed as assigned to etoposide following the intention-to-treat approach.

**Figure 1:**
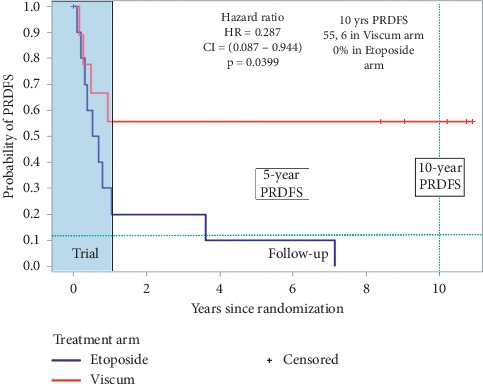
Postrelapse disease-free survival.

Due to inadequate recruitment compared to what it was planned (36 patients in three years, 18 each arm), the study was terminated early after 48 months by protocol amendment after the inclusion of 20 patients (11 etoposide and 9 *Viscum*). Data updated in July 2013 were already published [25]. Patients were continued to be followed periodically. The analysis presented here has been updated on June 30, 2019, 12 years after the start of the study and at a median follow-up of 106 months (range 3–144) of 19 treated patients or 83 months (3–144) considering intention-to-treat (ITT) analysis on 20 randomized patients. Males were 11 and females 9; mean age at baseline was 33.9 years (range 11–65). Arms were well balanced for risk factors such as site of relapse, number of lesions at the second relapse and intervals from the end of chemotherapy and the first and second relapse, and percentage of necrosis of primary tumor (necrosis >90% = good responders; necrosis ≤90% = poor responder) (Table 1). ABCB1 P-glycoprotein positivity was also evaluated on a metastatic specimen resected previously to treatment as a negative prognostic factor in osteosarcoma [26]. ABCB1 positivity was revealed in metastatic tissue in 2 out of 4 patients in the *Viscum* arm (the other 2 were PgP negative) and in 1 out of 4 patients in the etoposide arm. The one-year PRDFS and OS were already reported: only the 1-year PRDFS rate of the *Viscum* arm differed significantly from the historical 12% rate (*p*=0.004) [23]. After the end of study participation, two patients of the *Viscum* arm continued to use *Viscum* either for 12 months or at a 6-month on/off schedule, respectively. One patient of the etoposide arm after resection of lung metastases as the third relapse crossed over to *Viscum* and continued for 3 years spontaneously, and he is alive, free from disease. One patient in the etoposide arm after a third relapse of osteosarcoma (lung metastases, resected) developed a second cancer one year after (GIST of stomach) which was resected. The patient is alive and free of both cancer in June 2019. The 5-years PRDFS rate in the *Viscum* arm was 55.5% (95% CI 21–86%) and 9% (95% CI 0, 2–43%) in the etoposide arm. The 5-yr OS was 66.7% (95% CI: 28.2–87.8) in the *Viscum* arm vs 40% (95% CI: 12.3–67) in the etoposide arm. On 30 June 2019, five out of nine enrolled patients in the *Viscum* arm never relapsed since the start of protocol, while all patients in the etoposide arm relapsed; the median PRDFS was 106 months (range 2–144) in the *Viscum* arm versus a median PRDFS of 7 months (range 1–84 months) in the etoposide arm (hazard ratio HR = 0.287, 95% CI: 0.076–0.884, *χ*^2^ = 4.714, *p*=0.0299) (Figure 1).

In the etoposide arm, 4 out of 11 patients were alive and free of disease after a 3^rd^ relapse, median OS 43 months (range 3–134), while 6 out of 9 patients in the *Viscum* arm were alive with a median OS of 120 months (range 14–144).

**Figure 2:**
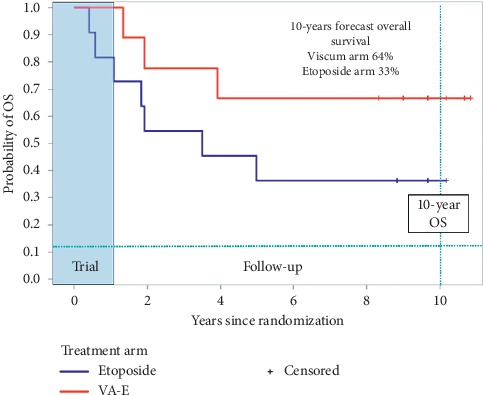
Overall survival.

A parametric proportional hazard regression model time forecasts 10-year OS rates for the *Viscum* and etoposide arms of 64% and 33%, respectively, assuming a lognormal distribution of survival (Figure 2).

On 30 June 2019, the PRDFS of the five long-term survivors in the *Viscum* arm were 144, 140, 134, 121, and 106 months, respectively.

**Figure 3:**
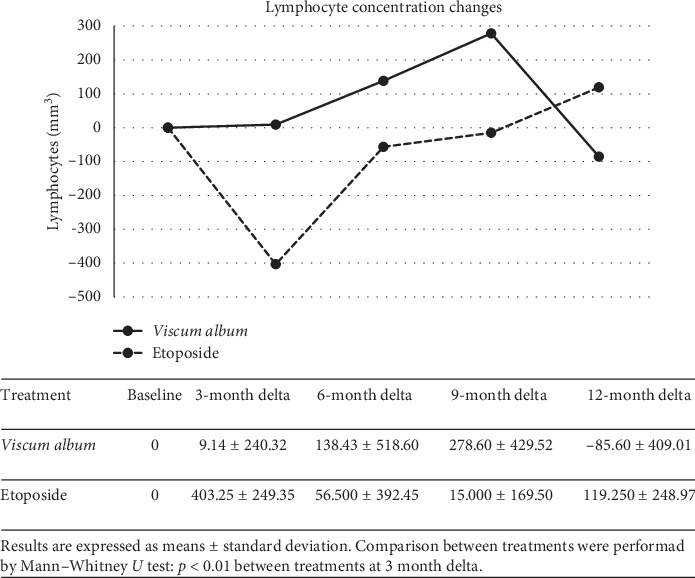
Total Lymphocyte count in Viscum- and Etoposide-treated patients at T0, T3, T6, T9, and T12.

**Figure 4:**
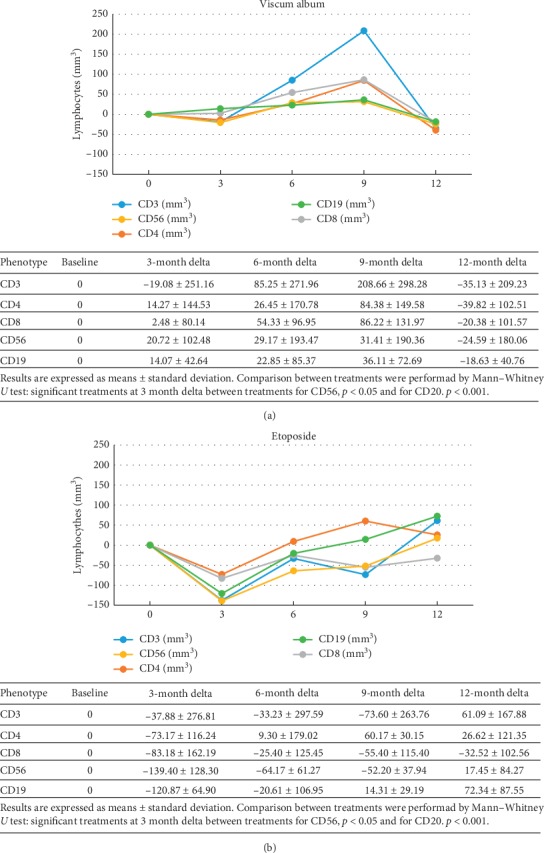
Lymphocyte subgroup count in (a) *Viscum* and (b) etoposide.

The count of lymphocyte subpopulations was performed in 7 patients in the *Viscum* arm (5 of whom had full test until T12 months) and 7 patients in the etoposide arm (4 until T12 months) at T0, T3 ms, T6 ms, T9 ms, and T12 ms, and they showed an increase in CD3, CD4, and NK after 6 ms of *Viscum* therapy, while a net decrease of these lymphocyte subpopulations was observed in the first 3 months of treatment in the etoposide group (Figures 3 and 4). The correlation between changes of each of the three T-lymphocytes subpopulations (CD3, CD4, and NK) demonstrated a consensual modification in the *Viscum* arm, while on the contrary, they had a disorganized pattern in the etoposide arm (Table 2). Patients after 12 months of study were continued to be followed with simple blood count regularly every visit, not with lymphocyte subpopulation count, and no abnormal changes were reported in total white blood cells or total lymphocytes in the following controls.

## 4. Discussion

The treatment of relapsed osteosarcoma patients remains unsatisfactory especially after a second and further relapse, and surgery is the best treatment when feasible and can prolong PRDFS (1, 2, and 3). Chemotherapy treatment in absence of measurable disease after complete surgical remission in relapsed patients is usually nonstandard. Few studies were published on maintenance therapy in osteosarcoma. A randomized study on metronomic chemotherapy as maintenance therapy in localized osteosarcoma was published in 2016. 298 patients with localized osteosarcoma were randomized to receive metronomic chemotherapy with oral cyclophosphamide + methotrexate for 73 weeks as maintenance therapy vs no therapy after usual neoadjuvant chemotherapy with MAP: no benefit on disease-free survival or overall survival was reported [27]. It is relevant that 35% of those patients randomized to receive metronomic chemotherapy refused it.

*Viscum album* fermentatum has a long history being used for over 80 years as a complementary integrative medicine, and it is safe with limited toxicity; it has been employed in several studies with some benefit reported on survival [28] and QOL, but many of these studies have methodologic flaws. *Viscum* has been employed intravenous [29], intratumoral [30], and also intravenously in pediatric cancer case reports. A recent case series from Essen pediatric oncology reported some benefit in heavily pretreated advanced pediatric cancer patients treated with intravenous *Viscum* (4/10 patients reported a partial response and 2/10 stable disease), and side effects requested hospitalization [31].

Twelve years after the start of our study, the patients treated with *Viscum album* fermentatum Pini continue to have a considerably and statistically significant longer PRDFS compared to the oral etoposide arm. Also, side effects and quality of life measured with EORTC QOL30 were favorable to the *Viscum* arm as previously reported [25]. *Viscum* can be administrated safely subcutaneously for years without serious side effects, while prolonged use of etoposide can increase the risk of second cancer. This study has the limitation of the small sample size. Patients were oligometastatic mainly to the lungs, so it was a favorable set of patients, but they were well balanced in the two arms, and the differences were significant. The 5-yr PRDSF results (55.5% in the *Viscum* arm) of this small study is better than previously reported [2, 4]. In fact, if we compare results from another report by Tirtei et al. [4], in which patients with lung metastases only have a 5-yrs post-second relapse DFS of 33.6% and those with one lung nodule only had a 5-yr PRDFS of 42% lower than the results of our study.

Also, the results of 10-year overall survival forecast are encouraging (64% for *Viscum* vs 33% for etoposide). Those patients who could reach a third complete surgical remission after the third relapse had an improved OS in both arms underling the importance of surgical resection of metastases to reach a longer survival. The analysis of lymphocyte subpopulation changes during time in the two treatment arms, indicating a different lymphocyte distribution between the two treatments, and at 6 months, it was almost opposite (*p* < 0,05) (Figure 3) with an increase of T subpopulations (CD3, CD4, and NK) in the *Viscum* arm, while a net decrease is observed in the etoposide arm (Figures 4(a) and 4(b)). *Viscum* could be used in patients free from disease at high risk of recurrence as maintenance therapy and compared in a randomized trial with other drugs or biologic response modifiers. If we consider that so far cytotoxic maintenance therapy has not proved effective in osteosarcoma, an inexpensive maintenance treatment like *Viscum* should be encouraged to be further evaluated in postrelapsed osteosarcoma.

So far, the scientific community and pharmaceutical companies showed no interest in randomized trials with *Viscum* compared to other conventional therapies. We hope that the results of this study will encourage future multicenter, international studies.

## Figures and Tables

**Table 1:** Patients characteristics.

PTS	Start TX	Age	Sex	Arm	Site/N°	T. necrosis	PgP on Met.	PRDFS	OS (ms)	Outcome	Status 30.6.19
1	08/06/07	48	F	Viscum P	Lung one	NA	NE	144	144	NED	NED
2	03/09/07	18	F	Viscum P	Lung two	PR	Pos	134	134	NED	NED
3	02/10/07	28	M	Viscum P	Lung two	GR	Pos	140	140	NED	NED
4	21/04/08	29	M	Viscum P	Lung one	GR	NA	3	17	R	Dead
5	19/09/08	30	F	Viscum P	Lung two	PR	NE	121	121	NED	NED
6	23/09/08	21	F	Viscum P	Lung three	PR	Neg	6	120	R	NED1
7	02/04/10	20	F	Viscum P	Bone one	PR	Neg	106	106	NED	NED
8	23/07/10	41	M	Viscum P	Lung one	GR	NE	2	24	R	Dead
9	30/06/11	22	M	Viscum P	Lung one	GR	Neg	11	36	R	Dead
10	12/06/07	28	M	Etoposide	Bone one	PR	NA	3	5	R	Dead
11	17/01/08	62	F	Etoposide	Lung one	GR	NA	12	128	R	NED 1
12	17/01/08	48	M	Etoposide	Lung one	PR	NE	6	134	R	NED 1
13	17/03/08	16	F	Etoposide	Lung one	GR	Neg	3	5	R	Dead
14	07/01/09	11	M	Etoposide	Lung two	GR	Neg	1	3	R	Dead
15	10/07/09	35	F	Etoposide	Lung one	NA	Neg	9	118	R	NED 1
16	28/05/09	65	M	Etoposide	Lung two	NA	Neg	84	119	R	NED 1
17	11/02/10	17	M	Etoposide	Lung two	PR	Pos	4	25	R	Dead
18	11/05/10	63	M	Etoposide	Lung one	NA	Pos	42	60	R	Dead
19	08/07/11	17	F	Etoposide	Lung two	PR	Neg	8	23	R	Dead
20	08/08/11	67	M	Etop never started ITT NA 43	NE	Dead	ITT				

Site/N° = site of the 2^nd^ metastase, N of lesions; tumor necrosis: necrosis on primary tumor; GR = good responder > 90% and PR = poor responder ≤ 90%; PgP on metastases: pos = Positive, Neg = Negative; NA = not available and NE = not evaluable; NED = nonevidence of disease after enrollment: NED1 = nonevidence of disease after treatment for a third relapse; R = relapse; ITT = intention-to-treat; PRDFS: postrelapse disease-free survival; OS: overall survival.

**Table 2:** Correlation between variations of cell population at different time points.

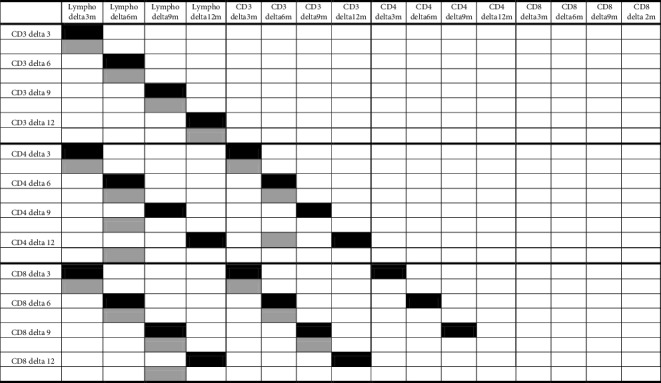

VA is reported in black, and Eto is reported in grey. Correlation was determined by the Pearson correlation test: *p* < 0.05 at least for all the colored boxes.

## Data Availability

Data regarding this study will be available if requested.
